# Illustrating the Multi-Faceted Dimensions of Group Therapy and Support for Cancer Patients

**DOI:** 10.3390/healthcare4030048

**Published:** 2016-08-01

**Authors:** Janine Giese-Davis, Yvonne Brandelli, Carol Kronenwetter, Mitch Golant, Matthew Cordova, Suzanne Twirbutt, Vickie Chang, Helena C. Kraemer, David Spiegel

**Affiliations:** 1 Department of Psychiatry and Behavioral Sciences, Stanford University, 401 Quarry Rd., Stanford, CA 94305, USA; stwirbutt@comcast.net (S.T.); vickieychang@gmail.com (V.C.); hckhome@pacbell.net (H.C.K.); dspiegel@stanford.edu (D.S.); 2 Department of Oncology, University of Calgary, 2202 2nd St. S.W., Calgary, AB T2S 3C1, Canada; ynbrande@ucalgary.ca; 3 Department of Psychosocial Resources, Tom Baker Cancer Centre, 2202 2nd St. S.W., Calgary, AB T2S 3C1, Canada; 4 California Pacific Medical Center, Breast Health Center, 3698 California Street, San Francisco, CA 94118, USA; kronenc47@gmail.com; 5 The Wellness Community/Cancer Support Community, Research & Training Institute, Cancer Support Community, 4100 Chamounix Drive, Philadelphia, PA 19131, USA; mitch@cancersupportcommunity.org; 6 Veterans Administration Medical Center Martinez, 150 Muir Rd., Martinez, CA 94553, USA; Matthew.Cordova@va.gov; 7 Pacific Graduate School of Psychology, Palo Alto University, 1791 Arastradero Road, Palo Alto, CA 94304-1337, USA

**Keywords:** emotional expression, cancer, support groups, community, psychotherapy process

## Abstract

In cancer support groups, choice of therapy model, leadership style, and format can impact patients’ experiences and outcomes. Methodologies that illustrate the complexity of patients’ group experiences might aid in choosing group style, or testing therapeutic mechanisms. We used this naturalistic study as a beginning step to explore methods for comparing cancer group contexts by first modifying a group-experience survey to be cancer-specific (Group Experience Questionnaire (GEQ)). Hypothesizing that therapist-led (TL) would differ from non-therapist-led (NTL), we explored the GEQ’s multiple dimensions. A total of 292 patients attending three types of groups completed it: 2 TL groups differing in therapy style ((1) Supportive-Expressive (SET); (2) The Wellness Community (TWC/CSC)); (3) a NTL group. Participants rated the importance of “Expressing True Feelings” and “Discussing Sexual Concerns” higher in TL than NTL groups and “Discussing Sexual Concerns” higher in SET than other groups. They rated “Developing a New Attitude” higher in TWC/CSC compared to NTL. In addition, we depict the constellation of group qualities using radar-charts to assist visualization. These charts facilitate a quick look at a therapy model’s strengths and weaknesses. Using a measure like the GEQ and this visualization technique could enable health-service decision making about choice of therapy model to offer.

## 1. Introduction

Investigation of the benefits of social support for disease-related stress is more than 30 years old [1,2]. Support groups are often effective in providing social and emotional support and a context for pertinent exchange of information for people with chronic or life-threatening illnesses [3]. These groups offer valuable additional support to that provided by health providers, partners, family, friends, and other members of patients’ social networks. Despite these advances, clinicians and program directors seeking to offer cancer support groups in a hospital or clinic have few tools to visualize the types of experiences available to their patients when they choose a particular group model. Support groups are multi-faceted and selection of therapy model, leadership style, and format may impact patient benefit [4,5,6].

Researchers have not created complex multi-dimensional visual representations of cancer support groups although they have created such methods for individual coping, personality (such as the Circumplex Model) [7,8], and emotional processing styles (Latency and Valence) [9]. Though scales measuring multi-dimensional aspects of groups exist [6,10], users may find it difficult to conceptualize the complexity of these dimensions as they relate to each other within theoretically distinct group therapy models.

While cancer patients can benefit from group support [11], not all support-group models effectively reduce distress, satisfy cancer patients, or produce long-term positive outcomes [12,13,14,15,16]. A program’s effectiveness in significantly decreasing mood disturbance [17,18], trauma symptoms [19], anxiety [20,21], pain [22,23], depression [21,24], and improving emotion-regulation [25], social functioning [21], adherence to treatment [21], and immune, cortisol, or cell-aging function [21,26,27,28,29] could depend on the choice of therapy model or a particular constellation of group dimensions or experiences. Because little process research examines moderating and mediating therapeutic mechanisms, we do not understand the reasons for these inconsistencies [30,31]. Existing cancer support groups vary in setting, format, leadership, therapeutic style, and emphasis, all of which could moderate or mediate outcomes [4,32,33,34]. A clear but comprehensive multi-dimensional model could inform decisions about type of support group to offer in a clinical setting. Additionally, standardized group measures that reflect therapeutic group process, may not reflect the breadth of dimensions that are meaningful to cancer patients participating in diverse group settings to give and receive support. These settings are broad and include therapist-led group therapy, but also include lecture-discussion, peer-led support, church, sewing, and social groups all providing some aspects of social support valued by patients. As an initial hypothesis-generating step toward this complexity, this paper presents a method to examine whether cancer patients value different aspects of their group experiences depending on variation in the group model, and then presents an illustration method for visualizing the overall experience of participants in several different groups attended by cancer patients.

This is a naturalistic study of participants already in group therapy or support. It is an examination of groups as they function and the experiences that participants using those groups found useful. We cannot disentangle the group from its membership or adjust for differing formats or participants in the current study as it is not a study of effectiveness and groups are not randomized. Our focus in this method demonstration is simply to highlight possible differences between groups and a novel visualization method to generate clearer directions for future studies.

Prior research on participants’ experiences in non-cancer support groups indicates that group environment may differ depending on leadership (professional vs. peer/member leaders) [35,36,37], therapy style, goals of the group [38], stage of the group [38,39,40], and individual differences in participants [39]. For cancer support groups, few studies report participants’ experiences, the process of these groups, and whether these experiences affect outcomes [32,33,41,42]. To our knowledge, validated measures of group processes specifically created for cancer support groups do not exist. In this paper, we used a measure created to allow patients to rate how much they valued specific experiences in the groups they attended that was modified to pertain to cancer support groups. To illustrate multi-dimensional support-group experiences, we compared here the most valued experiences of participants in three kinds of cancer support groups (Table 1) using convenience samples of groups we studied between 1994 and 2001 for exploration. Groups included two therapist-led cancer groups (TL) each differing in therapy style (Supportive-Expressive Group Therapy (SET), The Wellness Community (TWC/CSC)), and a range of non-therapist-led groups (NTL) attended by cancer patients that differed in format, leadership and goals (Self-Help Cancer Group, Lecture-Discussion Cancer Group, and Social Groups).

A common method to examine group differences would test these dimensions one at a time. Although researchers gain valuable information in this method, the overall constellation of these dimensions and the impact that the combination of dimensions might have on the patient are lost. A clinician’s sense of the total environment of each group style is fractured, and thus a sense of cancer patients’ holistic experience in these groups. Here, we present both common and novel methods as a demonstration, similar to the level of inquiry posited as phase Ia in a recent model of pilot discovery meant to lead to later randomized trials [43]. As a first step in developing this method, we examined in the traditional ways the dimensions and psychometrics of The Group Experiences Questionnaire (GEQ). Though definitive tests would require randomized trials and greater psychometric development, for this demonstration we explore here theorized differences between the two TL group styles. Because SET emphasizes emotional expression [25], we expected that participants would rate SET higher on “Expressing True Feelings”. Because TWC/CSC emphasizes cognitive approaches to becoming active in one’s recovery [44], we expected participants to rate it higher on “Developing a New Attitude”. Each of these expectations follows from explicit models of group therapy facilitation [44,45]. We then adapted an illustration method using radar charts, an excellent way to visualize multivariate complexity [46,47]. The radar chart displays data as a set of equi-angular spokes, or radii, where spokes characterize variables or processes [48,49]. When dimensions are ordered in theoretically meaningful ways, a viewer can see patterns among multiple dimensions for each group [47,49]. We used radar charts to explore and illustrate the extent to which each group style had its own constellation of important experiences. We also examined participants’ ratings of satisfaction. We expected that there would be no differences in satisfaction despite differences in what group experiences cancer patient value. Participants may benefit in fundamentally different ways, though report similar satisfaction. The current paper is an initial step toward a long-term goal to examine whether choices of group format, style, and leadership may directly affect the range of group experiences available to and valued by cancer patients.

## 2. Materials and Methods

### 2.1. Groups Studied

Women and men with cancer who selected to attend three different types of supportive groups completed the GEQ. This study was approved by the Stanford Institutional Review Board and all participants completed informed consent in accordance with the Declaration of Helsinki. Group and participant characteristics differ according to the setting (Table 1 and Table 2), and we cannot adjust for these differences in this naturalistic study.

#### 2.1.1. Stanford Supportive-Expressive Group Therapy (SET)

In SET [45], developed by Yalom from existential psychotherapy theory [50], therapists encourage open expression of primary negative affect (fear, direct anger, and sadness) [25]. Twenty metastatic breast cancer patients (of 47 possible randomized to the intervention) agreed to fill out the GEQ midstream in a clinical trial of SET (see [25] for complete description of this sample). Most of the 27 women who did not complete the questionnaire had died, or were too ill to complete an additional questionnaire. The current sample of 20 women did not differ from non-completers on demographic or medical variables.

#### 2.1.2. The Wellness Community (TWC) Now Called the Cancer Support Community (CSC)

Since 1982, TWC/CSC has offered a free program of psychological and emotional support for cancer patients and their families. Each week it serves over 5000 participants internationally, 600 within California, in professionally (licensed Social Workers, Psychologists, MFCCs) facilitated support groups. TWC/CSC trains therapists in their “Patient-Active” model that empowers patients to actively participate in their survivorship [44,51]. Participants in this study were members of on-going Participant Groups—weekly, committed, two-hour support groups for cancer patients with mixed diagnosis, gender, and age. The 159 women and 65 men who participated from TWC/CSC represented six sites in California, and all volunteered to complete questionnaires after a weekly group session during the same week.

#### 2.1.3. Non-Therapist-Led Groups (NTL)

We sampled a number of groups cancer patients attend for support that were not led by therapists (*n* = 48). These included a self-help cancer group (Our Cancer Support Group) (*N* = 15) created by patients and cancer specialists to meet patients’ needs. It was unstructured and open to men, women, spouses, and family members. The group elected naturally-skilled member leaders. We also included two lecture-discussion cancer groups (*N* = 12): One of them, Cansurmount, was a continuing-education and support group for an American Cancer Society peer-counselling program; the second is typical of many oncology-centre cancer support groups meeting in a clinic in the evening, where a variety of professionals gave informational lectures followed by patient discussion. Lastly, we included ratings of social groups attended by cancer patients (*N* = 21) for support during their recovery. These groups were attended by women with primary breast cancer who filled out questionnaires at baseline before randomization to different support group styles in a clinical trial [52,53]. The women included indicated that they participated in a social group that provided significant emotional support (book, church, anonymous, educational, sewing, work-related-support, and other health-related groups). If these women attended multiple such groups, we included in this analysis the group that they rated as the most supportive. We examined whether there were any significant differences on any of our subscales among the Non-Therapist-Led groups and finding none, collapsed those three into one group.

### 2.2. Measures

#### 2.2.1. Group Satisfaction Ratings

We asked participants to rate on a one-item, 0 (not satisfied) to 5 (intensely satisfied) Likert-type scale how satisfied they felt with their groups.

#### 2.2.2. Group Experiences Questionnaire Development (GEQ)

As an initial step, we created *The Group Experiences Questionnaire* [54] to reflect common experiences of cancer support groups by modifying scale structure and items generated initially by Lieberman [6], and modified by Roberts [55] for non-cancer groups. Our subscales reflect the clinical areas important to cancer patients and conform to some targeted areas for clinical intervention in cancer survivorship [56,57]: Expressing true feelings, discussing sexual concerns, developing a new attitude, establishing supportive contact, and accessing information and advice.

We modified a self-report scale (developed for non-cancer groups [6]) by altering wording and adding items to reflect common dimensions of cancer support groups as a beginning step in scale development. Cancer patients participating in support groups in Champaign-Urbana, IL, USA, and therapists and administrative staff involved in on-going cancer support group research in the Psychosocial Treatment Laboratory at Stanford generated an initial 25 items. Subjects rated each item on a Likert-type scale (0 (not applicable) to 5 (one of the two or three most important experiences)) for each item reflecting the importance of this aspect of the group.

Based on clinical theory, feedback from patients and previous research [58], and a heuristic principal components analysis, we constructed initial subscales, examined Cronbach’s alpha, and eliminated items that did not demonstrate adequate internal consistency, resulting in 23 items. This iterative process produced five subscales:

1. Expressing True Feelings (4 items): Expressing my true feelings; Talking about fears of death and suffering; Confronting difficult problems and fears; Getting honest feedback from others.
2. Discussing Sexual Concerns (1 item): Discussing Sexual Concerns.
3. Developing a New Attitude (6 items): Developing a new attitude toward life; Changing my behaviour in ways that feel satisfying; Gaining insight about myself; Owning up to maladjustment when it seems important; Learning that I am responsible for how I cope with my life; Becoming hopeful.
4. Accessing Resources and Advice (3 items): Gaining access to important information; Getting direct advice, suggestions, or education; Getting new understandings or explanations.
5. Establishing Supportive Contact (9 items): Talking about everyday things and socializing; Developing new friendships; Belonging to and being accepted by a group; Playing my part at the meeting; Being encouraged to talk more; Modelling myself after other group members; Getting support and encouragement; Helping others; Making contact with someone who I could call on for help.

### 2.3. Analysis

Using SPSS, first we examined one-week test-retest reliability for our scale using Spearman Rank correlations, as effect-size estimates using the TWC/CSC sample because it was largest. We also examined Cronbach’s Alpha for internal consistency in the TWC/CSC sample. Before comparing groups on subscales of the GEQ and the Group Ratings, we converted all data (across all participants rather than within groups) to ranked percentile scores. In this way, we standardized the distribution across groups allowing comparisons. We conducted Omnibus One-Way ANOVAs with three Groups (Stanford’s SET, TWC/CSC, NTL) and used Sheffe post hoc tests to examine specific differences by scale and Cohen’s d for each paired contrast to report effect sizes (0.20 small, 0.50 medium, 0.80 large) [59]. Because this demonstration did not include randomized groups, we could not account for many aspects of variance due to logistical differences between groups or any variance due to the influence of group members on each other within specific groups [60].

## 3. Results

### 3.1. Initial Psychometrics and Satisfaction

For our largest group (TWC/CSC), for each subscale we present Spearman Rank correlations for one-week test-retest reliability (range: *r* = 0.51–0.64), Cronbach’s Alpha (range: *r* = 0.66–0.84), and subscale inter-relationships (range: *r* = 0.09–0.63) (Table 3).

#### Satisfaction

We could demonstrate no significant Satisfaction group differences in an Omnibus ANOVA F (2, 267) = 1.11, *p* = 0.33 (Table 2).

### 3.2. GEQ Between-Group Analysis

#### 3.2.1. Expressing True Feelings

**Figure 1:**
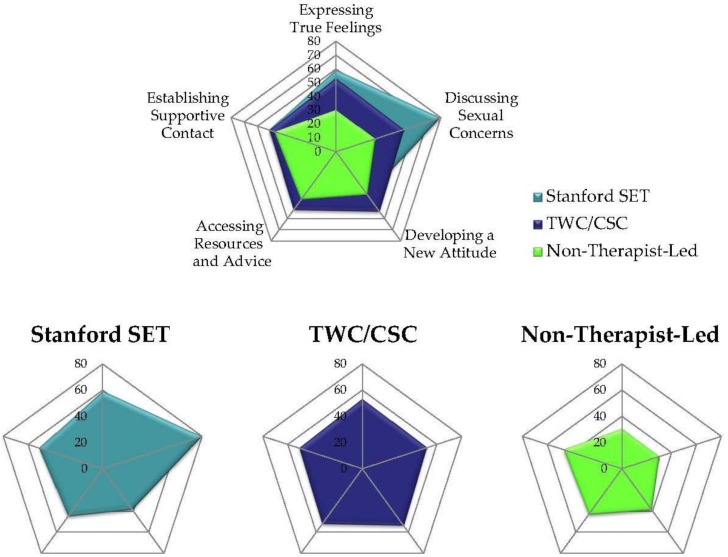
(**Top**) Overlapping radar plots of ranked percentile scores (across groups) of which group experiences were most important to participants (Group Experience Questionnaire) in 3 support groups by 5 subscales. Distance from center is level of ranking of importance by participants within each subscale (represented on the axes). Each color represents a different support group format. By examining shape, readers can perceive the constellation of experiences (subscales) most important within each group format. A particular subscale emphasis for a group can be seen where a color prominently extends beyond the colors for other groups outward on the axes; (**Bottom**) We present a separate radar chart for each group’s shape, since in the overlapping plot on top some points are occluded.

Groups differed significantly in an Omnibus ANOVA F (2, 292) = 15.08, *p* < 0.001). In Sheffe post hoc contrasts, participants rated both TL groups significantly higher than NTL groups, (*p* < 0.001), but we could not demonstrate that SET was rated significantly higher than TWC/CSC (Table 4 and Figure 1).

#### 3.2.2. Discussing Sexual Concerns

Groups differed significantly in an Omnibus ANOVA F (2, 287) = 27.59, *p* < 0.001. In Sheffe post hoc contrasts, participants rated both TL groups significantly higher than NTL groups, (*p* < 0.001), and rated SET significantly higher than TWC/CSC (*p* < 0.001) (Table 4 and Figure 1).

#### 3.2.3. Developing a New Attitude

Groups differed significantly in an Omnibus ANOVA F (2, 293) = 7.40, *p* < 0.001. In Sheffe post-hoc contrasts, participants rated TWC/CSC significantly higher than NTL groups, (*p* < 0.02), but we could demonstrate, neither that TWC/CSC was rated significantly higher than SET (*p* < 0.08), nor that SET was rated higher than the NTL groups (*p* < 0.94) (Table 4 and Figure 1).

#### 3.2.4. Accessing Resources and Advice

We could not demonstrate significant group differences in an Omnibus ANOVA F (2, 293) = 2.31, *p* = 0.10.

#### 3.2.5. Establishing Supportive Contact

We could not demonstrate significant group differences in an Omnibus ANOVA F (2, 290) = 0.31, *p* = 0.73*.*

### 3.3. Within-Group Analysis

We explored whether specific subscales of the GEQ were significantly correlated with Satisfaction. For SET, Satisfaction was significantly correlated only with Expressing True Feelings (*r* = 0.47, *p* = 0.04). For TWC/CSC, Satisfaction was significantly correlated with 4 of 5 subscales Expressing True Feelings (*r* = 0.25, *p* < 0.001), Discussing Sexual Concerns (*r* = 0.16, *p* = 0.02), Developing a New Attitude (*r* = 0.21, *p* = 0.002), and Establishing Supportive Contact (*r* = 0.31, *p* < 0.001). We could demonstrate no significant relationships between subscales and Satisfaction for the NTL Group.

The above demonstration analyses represent an initial naturalistic examination of three types of supportive groups attended by cancer patients and are typical of traditional tests in which groups are compared on each dimension. This provides an example of one outcome, but does not help the clinician or researcher visualize the array of dimensions that may exist within a single group style, or a sense of how the arrays compare with each other.

### 3.4. Radar Chart Profiles of Groups

We present radar chart profiles of the groups as a novel way for researchers and clinicians to picture differences in constellations of group experiences. In Figure 1, each colour represents a group. Figure 1 (top) presents overlapping profiles of each group. Figure 1 (bottom) allows the reader to examine the entire profile for each group because some of the shapes are occluded in the overlapping figure. In Figure 1, we have arranged the subscales around the axes so that dimensions that are theoretically part of TL compared with NTL groups are next to each other [47]. The two scales that are more emotionally focused are next to each other (Expressing True Feelings and Discussing Sexual Concerns), the two more cognitively focused are next to each other (Developing a New Attitude and Accessing Resources and Advice), and the one more socially oriented follows (Establishing Supportive Contact). In Figure 1, a reader can see a specific therapy or support group’s emphasis where the colour (or shade) extends prominently outside the bounds of the other groups. For instance, it is easy to see that SET’s emphasis on Expressing True Feelings and Discussing Sexual Concerns stands out in contrast with the other groups. In TWC/CSC, Developing a New Attitude is a unique aspect of their group model by comparison with the other groups. Participants in the NTL groups place more emphasis on the socially supportive aspects of their groups. We conclude that although TL and NTL supportive groups for cancer patients share some dimensions, none of these profile shapes are identical.

## 4. Discussion

As a clinical research community, it is clear that we do not understand well enough the core therapeutic mechanisms in group therapy and community support for cancer patients [4,31,34,61]. This study begins to define some important dimensions of these groups, and our illustration method offers clear evidence that these group environments are not alike for patients. We do not know whether these dimensions are related to group outcomes, although one recent study found that “disclosure” and “support” were related to lower depression symptoms in another TWC/CSC sample in cross-sectional analyses [32] and another that particular emotional expressions were related to decreasing depression and increasing social network size in SET groups [34]. The GEQ needs further validation, development, and testing including adding items to the Discussing Sexual Concerns scale. In addition, further work may validate or change the number or meaning of dimensions.

In the hypothesis-generating analyses for this study, we found support for our expectation that patients value expressing their emotions and discussing sexual concerns to a greater extent in TL than NTL groups. Therapists may be more likely to provide focus and greater safety for strong and intimate emotional exploration and expression. Expressing vulnerable feelings and discussing sexuality within a peer-led or a lecture-discussion group may feel too threatening. We also wonder whether the very high rating of importance in the all-female SET metastatic breast cancer group, where intimate and emotional issues are particularly emphasized, gives some indication of how important such discussions could be for cancer patients if their support-group norms allowed or encouraged them. We also found that TWC/CSC uniquely provided an environment in which the development of a new attitude was valued. This finding validates TWC/CSC’s stated goal of providing a clear emphasis on “The Patient-Active Concept”, encouraging greater personal responsibility for one’s care and choices as a cancer patient.

We found these differences in the importance of specific experiences in these support groups despite finding no difference in participants’ satisfaction. This indicates that the differences we demonstrate are not simply a matter of participants being less satisfied with their group experiences, but instead are likely to reflect the different range of experiences and discussions in different types of groups.

The types of group therapy and support offered in many hospital and community settings are often selected based on considerations other than evidence from randomized clinical trials (such as staff availability and time). Because many of these settings offer lecture-discussion formats, and/or groups that are not facilitated by therapists, future research might hypothesize that such environments are not likely to encourage discussion of emotional and intimate issues, and may not facilitate cognitive change. Though patients may express satisfaction with them, it is unclear how effective these environments would be in reducing distress or traumatic reactions which are outcomes associated with TL groups in prior studies [19].

NTL groups, however, offer valued social support, often normalize patients' reactions, help cancer patients create new social networks, and provide access to one of the most important aspects of all cancer groups--hearing other’s stories, symptoms, and treatment options. In the current study, all participants valued the social aspects of their group experience equally regardless of group format, leadership, or style. Research has not often examined the importance of social peer support groups. However, in interviews conducted by the first author with women in both the SET and NTL groups, they often said that access to discussions with peers was the primary reason that they sought support groups. Not surprisingly, they also said that attending NTL social groups allowed them to minimize the impact of their diagnosis and escape their cancer-patient role.

This study is limited by a cross-sectional design with unequal samples that utilized self-selected participants in differing settings and could not represent every type of group therapy and support for cancer patients known to be valuable or available [13]. As such, these participants likely differed in many ways that were uncontrolled by our research design, and we are limited in generalization to the groups and populations studied. We also cannot distinguish between whether these groups did not discuss the topics included in each dimension from whether those dimensions were just not important to the participants. We believe, however, that we provide important descriptive information and an interesting method for examining the holistic profiles of group experiences as a naturalistic observational study.

These methods may help clinicians understand what participants’ value in the groups they offer. In addition to graphing different types of support group styles, a clinician could give participants this questionnaire (or other scales using group dimensions) to begin to assess what aspects of group support are more important for an individual participant or certain types of individuals. Those most satisfied compared with least satisfied may appear to value different aspects of the groups offered [62]. Men compared with women may value a different constellation of group dimensions [35]. Repressors compared with high-anxious participants may also value a very different constellation of dimensions in a group. Individual participants could also indicate their ideal group experience ahead of being referred to a group in order to match preferences to the group contexts available. Lastly, examination of moderators and mediators of group benefit might be facilitated by this method [63]. For instance, in following a group of patients naturally attending varying support groups, it would be interesting to examine what happens over time for women with breast cancer attending groups where discussions of sexuality are not valued. Would their sexual function fail to improve over time compared with those attending groups where these discussions were routinely valued?

## 5. Conclusions

In this initial study demonstrating a method to distinguish patients’ experiences of cancer support groups, we found preliminary support demonstrating group differences that were in alignment with theoretical therapy models through the use of an initial version of a cancer-support-group specific group measure (GEQ). We presented an innovative graphic and these techniques to open opportunities for greater understanding of cancer patients’ experiences of group therapy and support. We look forward to further use of this graphic, and to further development of the GEQ or other dimensional group measures, so the experiences participants’ value in cancer support groups can be visualized easily.

## Figures and Tables

**Table 1:** Group characteristics for 3 types of supportive groups attended by cancer patients (*N* = 292).

Characteristic	Therapist-Led		Non-Therapist-Led
Group	Stanford SET (*n* = 20)	TWC/CSC (*n* = 224)	Self-Help, Lecture/Discussion, Social Groups (*n* = 48)
Setting	University Randomized Trial	Community Clinical setting	Community Hospital, ACS Cansurmount, and Community Randomized Trial
Location	S.F. Bay Area, CA	6 Sites in CA	Champaign-Urbana, IL and S.F. Bay Area
Format	Facilitated, Unstructured Emotional Support	Facilitated, Unstructured Emotional Support	Un-Facilitated, Unstructured Emotional Support, Structured Lectures/Discussion
Leadership Style	Supportive-Expressive	Patient-Active Concept	Peer-led, Lecture, and Un-led
Characteristic	Mean (SD)	Mean (SD)	Mean (SD)
Meeting Size	5.75 (2.24)	9.16 (2.40)	14.22 (12.47)
Meeting Length (h)	1.50 (0.0)	2.00 (0.0)	5.22 (13.94)
Meetings per Month	4.00 (0.0)	4.00 (0.0)	2.79 (4.50)
% Women	100	69.1	78.9
% Breast Cancer	100	23.9	30.3
% Metastatic	100	39.6	15.0

Note: Stanford Supportive-Expressive Group Therapy (SET), The Wellness Community (TWC/CSC: Currently named The Cancer Support Community), American Cancer Society (ACS), Cansurmount was a peer-counseling program sponsored by ACS.

**Table 2:** Participant characteristics for 3 types of supportive groups attended by cancer patients (*N* = 292).

Characteristic	Therapist-Led		Non-Therapist-Led
Group	Stanford SET (*n* = 20)	TWC/CSC (*n* = 224)	Self-Help, Lecture/Discussion, Social Groups (*n* = 48)
	Mean (SD) Range	Mean (SD) Range	Mean (SD) Range
Satisfaction (Mean Percentile Rank)	58.46 (30.33)	49.02 (26.98)	48.92 (27.53)
Age	56.77 (11.30) 36.05–73.52	56.62 (11.74) 27.30–85.28	56.13 (9.88) 28.91–77.06
Months in group	27.80 (15.32) 3.48–55.49	12.82 (16.95) 0.10–120.60	54.96 (53.42) 1.5–473.07
Years from original diagnosis	7.85 (3.27) 1.36–13.1	2.71 (4.55) 0.16–37.69	5.36 (4.77) 0.14–29.00

**Table 3:** Correlations among subscales, one-week test-retest reliabilities, and Cronbach Alphas for cancer patients in TWC/CSC Groups (*N* = 227).

Subscale	1 (*N*)	2 (*N*)	3 (*N*)	4 (*N*)	5 (*N*)
1. Expressing True Feelings	**0.55 (102)** **0.66 (220)**				
2. Discussing Sexual Concerns (single item)	0.32 (223)	**0.64 (102)**			
3. Developing a New Attitude	0.56 (227)	0.45 (223)	**0.51 (102)** **0.76 (216)**		
4. Accessing Resources and Advice	0.26 (227)	0.10 (223)	0.35 (227)	**0.55 (102)** **0.75 (224)**	
5. Establishing Supportive Contact	0.54 (226)	0.38 (223)	0.63 (226)	0.35 (226)	**0.64 (102)** **0.84 (209)**
6. Satisfaction	0.25 (207)	0.16 (204)	0.21 (208)	0.07 (207)	0.31 (206)

Note: Spearman Rank Order Correlations among subscales and one-week test-retest reliabilities (also Spearman Correlations) in bold in the diagonal. Cronbach’s Alpha for subscales in bold and underlined in the diagonal. The Wellness Community (TWC/CSC): Currently named The Cancer Support Community (CSC).

**Table 4:** ANOVA results comparing groups attended by cancer patients (*N* = 296) for group experience questionnaire subscale percentile ranks (across groups).

Mean Percentile Rank of Subscale	Therapist-Led		Non-Therapist-Led	*p* Value, d
	Stanford SET	TWC/CSC		
	(*n* = 20)	(*n* = 228)	(*n* = 48)	
	Mean (*SD*)	Mean (*SD*)	Mean (*SD*)	
Expressing True Feelings	59.56 (29.24)	53.49 (27.51)	30.54 (26.50)	0.001
SET vs. TWC/CSC				0.64 ^a^, 00.21
SET vs. NTL				0.001 ^b^, 1.04
TWC/CSC vs. NTL				0.001 ^c^, 0.85
Discussing Sexual Concerns	79.77 (19.81)	51.98 (26.10)	30.06 (26.55)	0.001
SET vs. TWC/CSC				0.001 ^a^, 1.20
SET vs. NTL				0.001 ^b^, 2.12
TWC/CSC vs. NTL				0.001 ^c^, 0.83
Developing a New Attitude	38.66 (27.79)	53.61 (27.75)	38.62 (30.35)	0.001
SET vs. TWC/CSC				0.08 ^a^, 0.65
SET vs. NTL				0.94 ^b^, 0.09
TWC/CSC vs. NTL				0.02 ^c^, 0.49
Accessing Resources and Advice	45.19 (28.72)	52.10 (28.10)	43.09 (30.20)	0.10
Establishing Supportive Contact	50.97 (24.89)	50.75 (28.70)	47.15 (31.55)	0.73

Note: Significance tests were two-tailed. Study variables are subscales of the Group Experience Questionnaire (GEQ). We used Sheffe post-hoc tests to adjust for multiple comparisons. a: SET vs. TWC/CSC; b: SET vs. Non-Therapist-Led (NTL); c: TWC/CSC vs. NTL; d: Cohen’s d effect size.

## Abbreviations

The following abbreviations are used in this manuscript:

TL	Therapist-led
SET	Supportive-Expressive Group Therapy
TWC	The Wellness Community
CSC	Cancer Support Community
NTL	Non-therapist-led
GEQ	Group Experience Questionnaire
MFCC	Marriage, Family, & Child Counsellor
